# HLA B27 allele types in homogeneous groups of juvenile idiopathic arthritis patients in Latvia

**DOI:** 10.1186/1546-0096-8-26

**Published:** 2010-10-14

**Authors:** Valda Stanevicha, Jelena Eglite, Dace Zavadska, Arturs Sochnevs, Arina Lazareva, Dinara Guseinova, Ruta Shantere, Dace Gardovska

**Affiliations:** 1 Department of Pediatrics, Riga Stradins University, Latvia Vienības gatve 45, Rīga, LV 1004, Latvia; 2 Laboratory of clinical immunology and immunogenetics, Riga Stradins University, Latvia Biķernieku iela 29- 30, Rīga, LV 1039, Latvia; 3 Laboratory of clinical immunology and immunogenetics, Riga Stradins University, Latvia Kr.Valdemāra iela 111- 3, Rīga, LV 1010, Latvia; 4 Department of Pediatrics, Riga Stradins University, Latvia Adrese: Aviācijas iela 5- 46, Rīga, LV 5- 46, Latvia; 5 Children University hospital, Department of Rheumatology Vienības gatve 45, Rīga, LV 1004, Latvia

## Abstract

Juvenile idiopathic arthritis (JIA) is a heterogeneous condition and therapeutic strategies vary in different JIA types. The routinely accepted practice to start with Sulphasalazine (SS) as the first line treatment in patients with HLA B27 positive JIA proves to be ineffective in a large proportion of children.

**Objective:**

to investigate HLA B27 positive JIA patients clinical characteristics, determined HLA B27 allele types and their connection with antirheumatic treatment in homogenous patient groups.

**Materials and methods:**

56 patients diagnosed with JIA and observed over the period 2006 to 2009 included in the study. HLAB27 allele types were determined using PCR method.

**Results:**

In HLA B27 positive JIA patients mean disease onset was 12.34 ± 3.3 years. Most common (44%) JIA type was enthesitis related arthritis. Positive response to the treatment with SS was found in 32% of patients, Methotrexate (MTX) - in 43%, combined treatment - SS with MTX was effective in 12.5%. 12.5% of patients required combination MTX with Enbrel.

Eight HLA B27 allele types were found in JIA patients in Latvia: *2702, *2703, *2704, *2705, *2710, *2715, *2717, *2728. The most common was *2705 - in 55% of cases. Among all the patients enthesitis related arthritis most commonly occurred in patients with HLAB*2705 allele (OR = 2.01, p < 0.02), oligoarthritis in patients with *2710 allele (OR = 3.0, p < 0.04) and polyarthritis with *2717 allele (OR = 3.0, p < 0.05). In patients with *2705 allele effective treatment was MTX (OR = 1.13, p < 0.03) and MTX with SS (OR = 2.02, p < 0.05), but in patients having *2703 allele - MTX with Enbrel (OR = 2.94, p < 0.02).

**Conclusions:**

There are 8 different HLA B27 alleles in JIA patients in Latvia and the most common is *2705, but in order to assert them to be disease associated alleles, more extensive studies are needed, including control group of HLA B27 positive healthy individuals. Standard treatment approach with SS proves to be unsatisfactory in the majority of JIA patients. To improve children's quality of life achieving rapid disease control, the first line treatment in HLA B27 positive patients should be MTX. In order to start with the most appropriate drug it is necessary to determine HLAB 27 type at the onset of disease.

## Introduction

Juvenile idiopathic arhtritis (JIA) is the most common chronic rheumatic juvenile disease, causing substantial invalidity, social and psychological problems both for the children and their parents. According to information sources the incidence of the disease is 1 - 22 per 100 000 children/year and prevalence 8 - 150 per 100 000 children [1].

Nearly half of the patients continue carrying the disease also in adult age [2]. Radiology tests have proved that bone damages among most of the patients with systemic or polyarticular arthritis, appear already within the first 2 years, and among patients with oligoarthritis - within 5 years of the disease. 50 - 70% of patients with systemic arthritis and 40 - 50% of patients with oligoarthritis in adult age, preserve the disease in its active phase. 30 - 40% of patients suffer from long-term joint dysfuncion , invalidity or physical incapacity, unemployment and 25 - 50% need surgical help, including joint prosthetics [3]. JIA extra-articular manifestations are uveitis, mainly iridocyclitis, which occurs in 21% of oligoathritis patients, and in 10% of patients with polyarthritis. Complications of uveitis are posterior sinechia, cataract, glaucoma and ocular disorders [1]. During last 15 years, the tactics of medical care of JIA patients has remarkably changed. The approach now is early and aggressive, based on disease-modifying medication to suppress the inflammation promptly and effectively. Thanks to this approach, the number of patients with functional disability has considerably decreased.

JIA is not a single disease, but a group of heterogeneous diseases that combines all kinds of chronic arthritis of unknown aetiology, which persist more than 6 weeks and the onset is before 16 years of age. Heterogeneity of JIA becomes apparent in many ways: in the onset of disease, in clinical features, symptoms, in the progress of disease as well as in genetic basis.

According to ILAR (*International League of Associations for Rheumatology*) classification, JIA has 7 disease types [4].

Up to now origin of the disease is unclear, pathogenesis mechanism of the disease is still under research, but what matters are environmental factors (psychological stress, traumas, injuries, viral and bacterial infections) as well as different genes, which determine a person's characteristic respondent (counter-action) features to immunity and inflammation. Several studies have proved the importance of certain genes being a risk factor in the development of the disease. One of the most investigated genetic associations with certain rheumatic diseases is HLA alleles, which were first described already in 1973, when it was proved that there is an association between HLA B27 allele and ankylosing spondylitis (AS) among adults [5,6]. Approximately 95% of AS patients carry HLA B27, at the same time in general population this allele is present only in 10% of European inhabitants, 1% in Japan, and the greatest occurrence is among native Americans (10 - 50%) [5,7]. It is documented that JIA with oligoarthritis has a positive association with several HLA class I un II gene alleles: HLA-A2, HLA-DRB1*11 (HLA-DR5 subtype) and HLA-DRB1*08. JIA with positive rheumatoid factor (RF) is considered to be an equivalent of adult RF positive rheumatoid arthritis (RA) and like for adults is associated with HLA-DR4. JIA type with negative RF is heterogeneous and HLA associations are not so distinct as of other types. Arthritis with enthesitis is associated with the presence of HLA B27 allele [8]. Arthritis with enthesitis is a disease, similar to spondyloarthropathies, but children, unlike adults for whom the dominating symptom is inflammatory backache, have their extra-axial joints involved in the process - mainly lower extremities. For some patients with arthritis with enthesitis the disease may advance and affect sacroiliac joints and spine, and within 5 -10 years it may develop AS clinical symptoms, however usually it takes many years before sacroilitis and spondylitis get visible radiologically [9]. It is known that in children HLA B27 alleles are connected with other JIA types, like oligo- and polyarthritis, but especially with arthritis with enthesitis and juvenile spondyloarthropathies [10-12].

Human leukocyte antigen HLA (Human Leukocyte Antigen) B27 or allele HLA B27 is a cell-surface protein and genetically the main cell compatibility complex MHC class I molecule [13]. It possesses several proven specific functions within immune system, but many functions stay still unclear. One of the main functions of this molecule is ability to present peptide antigens, which result from intracellular degradation of proteins, to other cells of the immune system, mainly to CD8 T cells. Recent researches revealed the importance of this molecule in the function of natural killer cells [14].

It is found out, that HLA B27 is not a single allele, but it represents a highly homologous HLA B alleles family, called types of alleles and denoted as HLA B*2701, B*2702 etc. The number of alleles discovered is constantly growing and up to now there are already 31 types: from B*2701 to B*2727, and moreover - allele *2705 has five subtypes - *270502-270507 [15]. These alleles differ from each other by one or several amino acids in peptide binding site, and by their diverse race/ethnic prevalence in the world population [16].

In Latvia according to data of the Latvian Rheumatic Disease Patient Registry, in 2006 the JIA prevalence was 861 patients, among which 119 carried positive HLA B27 allele. Each of HLA B27 positive patients may have a different type of disease (polyarhritis, oligoarthritis, arthritis with enthesitis), as well as a different degree of disease activity. Usually the presence of this allele determines the primary choice of therapy - treatment with Sulphasalazine (SS). SS is one of the first anti-rheumatic drugs and the first drug that has been developed specially for RA treatment, today SS is considered as DMARDS group drug. In 1930ies Prof. *Nanna Svartz*, a rheumatologist at the Karolinska Institute in Stockholm, advanced a postulate that RA is a disease of bacterial aetiology and it should be treated with preparations of sulphonamide group, which at that were already available. Prof. *Svartz *noticed that in RA treatment, combining the antibiotic sulphanilamide with the salicylates of anti-inflammatory therapy did not bring any solid benefit, and produced significant gastrointestinal side effects. *Svartz *was of an opinion, that these two medical products when combined chemically could at least decrease the gastrointestinal intolerance and asked Swedish Pharmaceutical enterprise Pharmacia in Upsala to merge chemically these two preparations. Thus linking sulphapyiridine to 5-aminosalicyilic acid, a new medication was derived with the name Sulphasalazine [17]. First reports on using SS in JIA therapy appeared in 1986, when *Özdogan *in his research work published the results on 18 JIA patients [18]. Since then the efficacy of SS in JIA therapy was proved in several other studies [19-29].

In rheumatology efficacy of the therapy can be judged in 3- 6 months after the beginning of the treatment. *Van Rossum et al *in double-blind placebo-controlled trial showed, that SS is a safe and effective medication both managing the symptoms of arthritis, and improving the inflammation data in patients with oligo- and polyarticular JIA, though one third of the patients developed intolerance [19,28]. SS is indicated also for patients with HLA B27 associated spondiloarthropathies [24]. Exact mechanism of SS performance is still not fully clear and is under continuous research. It is known, that SS has several anti-inflammation and immunomodulating effects: inhibits cell chemotaxis in inflammation, separation of mononuclear cell cytokines, proliferation and activation of lymphocytes, formation of osteoclasts, angiogenesis, activity of folate-dependent enzymes. Primary side-effects which may occur during SS therapy are gastrointestinal problems (anorexia, nausea, diarrhea), rashes, headache and increase of liver transaminases.

The clinical praxis shows that, SS therapy efficacy may vary with different JIA patients: in one part of patients SS takes a good control over the progress of arthritis, as well as decreases laboratory figures of inflammation, in other part of patients the SS efficacy is insufficient and it is necessary to combine or change the medication. Patients with insufficient therapeutic effect to SS, receive instead a different basic drug - Methotrexate (MTX). In severe cases of disease, combined therapy of MTX and SS is prescribed. If such conventional therapy however proves to be ineffective, the patient receives treatment with glycocorticosteroids or biological medication, like, TNF alpha receptor blocker - Etanercept.

Unfortunately, so far there is no scientifically grounded explanation found for such differences. There are no studies done yet in children rheumatology regarding MHC and choice of JIA treatment. Probably because the research of different JIA subtypes and associated genes are still in progress and the ILAR classification continues its improvement [30]. Another fields of medicine already carry out active pharmacogenetic research in treatment of different diseases, as well there is already the practical use in patients therapy , for example in HIV patients[31,32], hepatitis C in field of hepatology [33], in treatment of multiple sclerosis in the field of neurology [34,35]. There are few articles in rheumatology on individual HLA allele association with choice of therapeutical drugs, e.g. HLA B27 positive heel enthesitis treatment with Adalilumab [36] or HLA DR3 positive rheumatoid arthritis treatment and side effects with gold therapy [37].

Taking into account, that JIA is a heterogeneous group of diseases, the therapy tactics for different types of the disease may differ. If it were possible already in early stage of the disease to predict, for which HLA B27 positive patients the SS therapy would be effective, and for which patients the therapy would not achieve the desired efficacy, then patients could be prescribed the most appropriate and effective medication from the very beginning, to get early control of the disease, because, as studies of long-term observations show, suppressing the activity of the disease already at the early stage of the disease, has a favourable influence on the further progress of the disease, and the positive therapeutic effect can persist in a longer period of time [19].

In Latvia for children with JIA no genetic studies have been performed on the incidence of allele types HLAB27, as well as their possible connection with different JIA types and choice of therapy.

Hereby, hypothesis is raised, that there might exist connection between allele types HLA B27, JIA types and patient's reaction to anti-rheumatic therapy.

## Materials and methods

### Subjects

The study design was compound - prospective and retrospective, where during the period from year 2006 - 2009, in the study were included 56 children at the age from 3 to 18 years. All patients had HLA B27 positive JIA.

The JIA diagnosis and type of the disease was confirmed according to ILAR criteria. Efficacy of the treatment was evaluated and measured for every 3 month according to ACR Pediatric 30 a clinical response and improvement criteria [20,21]. The ACR Pediatric 30 use the following 6 basic a clinical response and improvement criteria: physician global assessment of overall disease activity; parent or patient global assessment of overall well-being; functional ability; number of joints with active arthritis; number of joints with limited range of motion and erythrocyte sedimentation rate. To consider a therapeutic response in JIA patients, improvement has to be at least in 3 of 6 criteria for 30% [21].

HLA class I HLAB27 allele was determined by polymerase chain reaction (PCR). There was determined 31 type of HLA B27 (from B*2701-B*2727; *2705 type 5 sybtypes - *270502-270507).

### DNA isolation and HLA B27 typing by PCR

The DNA samples were taken starting with peripheral blood cells using the "salting-out" method [38,39]. The genetic amplification was performed through PCR [38,39]. MHC classes I antigens subtype identifying by PCR-SSP method that apply MHC class I allele: HLA-B*2701 to HLA-B *2727 specificity detection. These DNA samples were processed by SSP DYNAL Invitrogen Corporation USA.

DNA was extracted from whole blood using a "salting-out" technique. HLA-B loci were typed according to the DNA typing method described by Bunce et al [40]. This polymerase chain reaction (PCR) technique uses sequence-specific primer (SSP) reactions to simultaneously identify alleles of the class I locus in an allele-specific or group-specific manner.

To identify class I alleles each PCR reaction consisted of in final concentration, 0.02 _g DNA, 0.42 units Taq DNA polymerase, 1_L of 10X Buffer IV, 3.4 mM MgCl2, 250_M of each dNTP, 0.54 _L glycerol, 0.01 _L 100 mg/mL Cresol Red, and 5 _L of the allele-specific and control primer mixes at a concentration of 1-4 _M each. The PCR reaction mix was plated out into 96-well plates, and amplifications were carried. The cycling parameters used were as follows: 96°C for 1 minute, followed by 14 cycles of 96°C for 30 seconds, 65°C for 50 seconds, and 72°C for 20 seconds, followed by 25 cycles of 96°C for 30 seconds, 62°C for 50 seconds, 72°C for 20 seconds, followed by 72°C for 4 minutes. The PCR products were electrophoresed in a 1.5% agarose gel for approximately 35 minutes at 150 V in 0.5 Tris-borate-EDTA (TBE) and visualized using UV illumination.

### Statistical

The HLA-B*2701 to HLA-B *2727 allele frequencies in patients with juvenile idiopathic arthritis and control subjects were compared using the chi-square (χ^2^) test and gene frequency (gf). The *P *value and odds ratio (OR) were calculated using EPI INFO software, version 06, with 95% confidence intervals and Fisher exact correction for small numbers [41].

## Results

The study included 56 white children - 25 boys (45%) and 31 girls (55%). Patient mean age was 12.34 ± 3.32 years (from 3 to 16 years). At the onset of disease 7% of patients were under 7 years and over 7 years -93%.

In the process of evaluation for received therapy we found 24 patients (43%) to be effective with MTX, 18 patients (32%) with SS. 7 patients (12.5%) required combined SS with MTX therapy, but in 7 patients (12.5%) due to ineffective standard therapy treatment with biological drug Etanercept with MTX were started.

### Division of HLA B27 positive JIA patient in groups

According to ILAR criteria all JIA patients were divided in 3 groups: 23% of oligoarthritis, 33%- polyarthritis seronegative and 44%- enthesitis related arthritis.

### Division of HLA B27 positive JIA patient by sex

In 96% of HLA B27 positive JIA boys was diagnosed enthesitis related arthritis and in 4% - polyarthritis. There were no oligoarthritis in boys diagnosed.

In 58% of HLA B27 positive JIA girls was diagnosed polyarthritis, in 39% oligoarthritis, but enthesitis related arthritis in 3%.

### Distribution of HLA B 27 allele types in homogeneous JIA patient groups

In JIA patients by typing HLA class I B27 with PCR *B2705 type (OR = 2.01, p < 0.02) was found more frequently (55%) in enthesitis related arthritis group, *B2710 (OR = 3.0, p < 0.04) in oligoarthritis group (11%) and *B2717 (OR = 3.0, p < 0.05) in polyarthritis patient group (9%). Other HLA B27 types were found respectively less (Table 1).

**Table 1:** Association between JIA type and HLAB27 allele type.

JIA subgroups	**Arthritis and enthesitis n = 25, *OR/χ***^***2***^***/p***	**Oligoarthritis n = 12, *OR/χ***^***2***^***/p***	**Polyarthritis, n= 19, *OR/χ***^***2***^***/p***	All children with JIA n = 56 , *gf*
				
HLA B*27 subtypes				
*2702	0.04	-	0.16	0.07

*2703	0.08	-	-	0.05

*2704	-	0.08	-	0.03

*2705	**2.01/5,25/0.02**	0.39/0,021**	0.39/0,021**	0.56

*2710	0.04	**3.0/0.710/0.04**	0.11	0.03

*2715	-	0.08	0.05	0.07

*2717	0.04	-	**3.0/0.81/0,05**	0.07

*2728	0.08	-	0.11	0.10

* HLA B*27 subtypes

Group JIA - children with juvenile idiopathic arthritis

** type protective statistically significant association for patients all JIA subjects

Results are only shown for those alleles where there was evidence for a difference between JIA subgroups.

Bold-face type highlights statistically significant associations for patients all JIA subjects;

*gf *(allele frequency), P (probability), OR (odds ratio) and x^2 ^values of OR are reported only for significant associations ( P < 0.05)

n = number of patients

### Distribution of HLA B 27 allele types by sex

By analyzing the frequency of HLA B27 allele types between male and female, the most frequently (68%) found type in boys was *B2705, but *B2703 type in 12% of cases and just in boys.

In girls the most frequently found HLA B27 allele type was *B2705 - in 45% of all female patients, but *2704 type in 3% of cases and *B2715 in 6% of cases and was diagnosed just in girls.

### Association of HLA B 27 allele types with ANA

In HLA B27 positive patients positive ANA was found in 18 cases (32%) with *2715 type (OR = 2.18, p < 0.05), but significantly less positive ANA cases in patients with *2702 (OR = 0.69, p < 0.01) type and *2717 (OR = 0.21, p < 0.00) type (Table 2).

**Table 2:** Association between ANA and HLAB 27allele type.

	All children with JIA, n = 56			
			
HLA B*27 subtypes	ANA positive n = 18	ANA negative n = 38	OR	*p *<
*2702	1	3	0.69**	0.01

*2703	1	2	1.06	0.09

*2704	1	-	nd	-

*2705	11	20	1.41	0.59

*2710	1	1	0.39	0.01

*2715	1	1	**2.18**	0.05

*2717	-	4	0.21**	0.00

*2728	2	3	1.46	0.28

* HLA B*27 subtypes

JIA - children with juvenile idiopathic arthritis

** type protective statistically significant association for patients all JIA subjects

Results are only shown for those alleles where there was evidence for a difference between JIA subgroups;

Bold-face type highlights statistically significant associations for patients all JIA subjects;

*gf *(allele frequency), P (probability), OR (odds ratio) and x^2 ^values of OR are reported only for significant associations ( P < 0.05)

n - number of patients

nd - not defined

### Occurrence of HLA B 27 allele types in homogeneous JIA therapy groups

In evaluation the efficacy of treatment in JIA groups and HLA B27 types, the data showed statisticcaly significant therapeutic effect of MTX (OR = 1.13, p < 0.03) and MTX with SS (OR = 2.02, p < 0.05) in *2705 type positive patients, but MTX with Etanercept combined therapy was necessary for *2703 type (OR = 2.94, p < 0.02) (Table 3).

**Table 3:** Association between effective treatment and HLAB27 allele type

Treatment Groups	**SS+ MTX n = 7, *OR/χ***^***2***^***/p***	**MTX n = 24, OR/χ**^**2**^**/p**	**SS n = 18, *OR/χ***^***2***^***/p***	**MTX+ Enbrel n = 7, OR/χ**^**2**^**/p**	All children with JIA n = 56 , *gf*
					
HLA B*27 subtypes					
*2702	-	0.57/0.13/0.01**	2.26/1.75/0.65	-	0.07

*2703	-	0.08	-	**2.94/0.50/0.02**	0.05

*2704	-	-	3.24/0.38/0.22	-	0.02

*2705	**2.02/0.16/0.05**	**1.13/0.54/0.03**	0.05	0.6/0.35/0.02**	0.55

*2710	0.29	0.08	0.49/0.35/0.05**	0.14	0.11

*2715	-	0.04	3.24/0.38/0.22	-	0.04

*2717	-	0.08	0.06	0.14	0.07

*2728	-	0.08	0.11	0.14	0.10

* HLA B*27 subtypes

JIA - children with juvenile idiopathic arthritis

** type protective statistically significant association for patients all JIA subjects

Results are only shown for those alleles where there was evidence for a difference between JIA subgroups.

Group SS +MTX indicates combination therapy with Sulfasalazine and Methotrexate

Group MTX indicates therapy with Methotrexate;

Group MTX+Enbrel indicate combination therapy with Methotrexate and Enbrel;

Bold-face type highlights statistically significant associations for patients all JIA subjects;

gf (allele frequency), P (probability), OR (odds ratio) and x^2 ^values of OR are reported only for significant associations ( P < 0.05)

n = number of patients

Therapy with SS, which is recommended start therapy in HLA B27 positive JIA patients, did not show significantly positive efficacy of treatment in any of HLAB27 allele type.

## Discussion

The present study in Latvia is the first of this kind regarding analysis of HLA B27 allele types, DMARDS therapy and their significance in children with JIA. Genetic studies are of significant importance, in order to comprehend the etiology and pathogenesis of JIA, as well will directly address the question of heterogeneity by analysis of the correlation between different JIA typs genomic variants and clinical responses to therapy. Different studies have shown that there exists association between HLA genes and certain JIA types [8]. *Murray et al *having studied HLA data in 680 JIA patients, came to a conclusion, that there exists a HLA specific age of years regarding onset of the disease, and it is unique for every JIA type [42]. In 1973 it was suggested that there was association between incidence of AS and HLA B27. Since then onwards, a great amount of scientific research has been performed on HLA B27, but no explicit knowledge of the role of this antigen in the disease pathogenesis, has been found up till now [6]. To date it has been found that HLA B27 is a heterogeneous allele group.

According to the published data, the most common HLA B27 allele types are B*2705 (in representatives of Caucasian race and American Indians), B*2704 (Asiatic), and HLA-B*2702 (Mediterranean population) [43]. All these alleles are linked also with AS. It has been found out that there are also other HLA B27 allele types associated with AS, like HLA-B*2701, B*2703, B*2707, B*2708, B*2710, B*2713, B*2714, B*2715, B*2719, and B*2725 [23]. The incidence of the largest part of these other alleles is small and rare, and their possible linkage with the disease, is not yet found out completely. It has been suggested, that there are 2 alleles, which do not have any connection with AS progression - HLA-B*2706, prevailing in Southeast Asia, and HLA-B*2709, which is found to be a prevailing allele on the Italian island Sardinia [44]. The two above mentioned alleles have not been detected in JIA patients in Latvia.

It is known, that HLA B27 allele has its role also in the paediatric population. According to the published data, 76% of children with arthritis and enthesitis have HLA B27 positive [45]. This type of JIA is associated with enthesitis and arthritis, and affects mainly boys after 6 years of age. In neighbour country Estonia, 28.6% of children with JIA, had positive HLA B27 [46]. There are 13, 8% B27 positive JIA patients in Latvia. *Bersntson et al *prospective studies with 305 JIA patients from three Nordic countries (Sweden, Norway and Denmark) concluded that HLA B27 is of great significance for boys with JIA. For HLA B27 positive boys, unlike patients with negative HLA B27 allele, it is typical that JIA is more active during the first three years of affection, with involvement of lower extremity joints [47].

According to the data of our studies in Latvia, boys and girls, primarily after 7 years of age, equally often develop HLA B27 positive JIA, the most frequent JIA type being arthritis with enthesitis (44%). However, analysing the JIA type incidence among boys and girls, it is the boys after 7 years of age, who most often develop arthritis and enthesitis (96%), whereas girls - polyarthritis (58%) and oligoarthritis (39%). Also other published research data confirm these correlations [5].

Totally it was detected, that in Latvia in children with HLA B27 positive, JIA is associated with 8 HLA B27 allele types: *2702, *2703, *2704, *2705, *2710, *2715, *2717, *2728, however, credibly the most frequent types are HLA B*2705,*2710 and *2717. Analyzing the data about relationship between JIA type and HLA B27 allele type, credibly positive association has arthritis with enthesitis with *2705 type (OR = 2.01, p < 0.02), polyarthritis with *2717 (OR = 3.0, p < 0.05) and oligoarthritis with *2710 type (OR = 3.0, p < 0.04). These findings once again show, that according to the published data, JIA is a heterogeneous disease and genetic predisposition varies with different JIA types [17].

There exists HLA B27 association with acute anterior uveitis (AAU) [48]. In our study uveitis was diagnosed in 3 patients and all three patients had *2705 type. AAU involves serious complications like glaucoma, cataract, ocular hypotony, formation of synechia, and consequently may lead to visual impairment and deterioration in the quality of life [49]. Frequently AAU progress may be asymptomatic, for that reason a timely diagnostics and treatment are of utmost importance in prevention of complications. The number of patients with uveitis involved in this study is insufficient to be able to develop any recommendations, but we believe that patients with *B2705 type need more frequent slit lamp examinations at oculist's.

JIA studies provide convincing evidence that there is genetic component and much of the genetic work, done in last three decades, related HLA genes' research, e.g. HLA class I B27 allele association with different JIA types and consequences [30,37]. Probably different JIA types and variable expression of drug receptors, metabolizing enzymes, and transporter systems have been linked to genetic polymorphism and individual differences in efficacy of DMARDS group drugs [35].

Today MTX is considered to be basic medication from among DMARDs in JIA therapy [50], and during our study, when treating with MTX, therapeutic effect was reached in 43% of JIA patients. But guidelines of JIA therapy recommend, that treatment of HLAB27 positive JIA cases should be started with SS, but evaluating the efficacy of treatment according ACR Pediatric30 criteria, our findings revealed, that with SS therapeutic effect was reached only in 32% cases, and still 12.5% of patients needed SS combination with MTX, but for 55.5% of patients it was necessary to remove SS, and therapeutic effect was reached only by MTX or MTX in combination with Etanercept.

Further comparing efficacy of treatment in homogeneous JIA type and HLA B27 allele type groups, it was found, that boys after age of 7, who most frequently developed arthritis and enthesitis and who credibly most often had *2705 type after 3 months of insufficient efficacy of treatment with SS, produced therapeutic efficacy with MTX and SS with MTX.

Analyzing separately the girl group we found out that also for girls, who in 3% cases developed arthritis and enthesitis and where also *2705 was present, credible therapeutic effect could be reached with SS with MTX, but for girls, in cases with association of *2710, SS does not produce any therapeutic effect. Likewise in cases of polyarthritis which girls had most frequently and association with *2717 was found, credibly effective DMARDs therapy was not proved.

Patients with *2703 need combined therapy - biological with MTX.

The obtained data of the efficacy evaluation suggest that the conventional primary JIA therapy with SS does not produce the desired therapeutic effect, and in order to keep control of the disease and to improve the quality of life for children, MTX should be chosen as primary therapy. However, in order to apply the most appropriate therapy from the early onset of the disease, it would be really necessary to determine the HLA B27 allele type, and aimfully launch a therapy with MTX, SS, MTX with SS or MTX with Enbrel. Moreover, to make the obtained data more exact and credible, it would be necessary to survey all HLAB27 positive JIA patients in Latvia.

Analyzing ANA connection with HLA B27 allele types and therapy efficacy in homogeneous JIA type groups, no credibility were found, so we may assume, that ANA most probably indicates the prognosis of the disease and not disease severity, like it was already proved in rheumatology with other autoimmune diseases, when, for instance, autoantibody - positive RF indicates to a prognosis of worsening and faster progressing course of disease in a case of seropositve JIA [10].

The study shows again, that JIA is a group of heterogeneous diseases with diverse genetic basis. It explains also the fact, that patients with different JIA types have varied reaction to the therapy. It would be very useful to continue genetic studies on JIA and HLAB27 allele types, because possibly, nuzzling of the genetic material is a way to finding the most suitable therapy, which, in its turn, is a key to more favourable prognosis of the disease.

## Conclusions

Our findings indicate a trend to have HLAB 27 positive JIA in children over 7 years of age and the most common type is enthesitis related arthritis. Girls tend to have HLA B27 positive oligo- and polyarthritis, where boys most often have enthesitis related arthritis.

In HLA B27 positive JIA patients the most effective disease modifying antirheumatic drug is MTX - in 43% of study patients, SS - 32%, but SS with MTX - 12.5% study patients. Combined biologic therapy with MTX and Etanercept was necessary for 12.5% of patients.

There are 8 different HLA B27 alleles in JIA patients in Latvia: *2702, *2703, *2704, *2705, *2710, *2715, *2717, *2728, and the most common significantly are *2705, *2710, *2717.

HLA B*2705 allele is significantly often found to enthesitis related arthritis group, *2710 in oligoarthritis group and *2717 - in polyarthritis patient group.

The data showed statisticcaly significant efficacy of treatment of MTX and MTX with SS in *2705 allele type patients, but MTX with Etanercept was effective in *2703 allele type patients.

Standard treatment approach with SS proves to be satisfactory in the half of HLA B27 positive JIA patients. To improve children's quality of life achieving rapid disease control, the first line treatment in HLA B27 positive patients should be MTX. In order to start with the most appropriate drug: MTX, SS, MTX with SS or MTX with Enbrel, it is necessary to determine HLAB 27 type at the onset of disease. But in order to assert the above mentioned data, more extensive studies are needed, including all HLA B27 positive JIA patients in Latvia which would lead to the development of good practice guidelines.

## Abbreviations

JIA: juvenile idiopathic arthritis; AS: ankylosing spondylitis; RF: rheumatoid factor; RA: rheumatoid arthritis; HLA: Human Leukocyte Antigen; MTX: methotrexate; SS: sulphasalazine; ILAR: International League of Associations for Rheumatology; ACR Pediatric 30: American College of Rheumatology (ACR) paediatric 30 definitions; OR: odds ratio; PCR: polymerase chain reaction; PCR -SSP: polymerase chain reaction with amplification with sequence-specific primers; DMARD: Disease Modifying Antirheumatic Drug; AAU: acute anterior uveitis

## Authors' contributions

SV has made substantial contributions to conception and design, acquisition of data, analysis and interpretation of data, participated in its design and coordination has been involved in drafting the manuscript or revising it critically for important intellectual content; and has given final approval of the version to be published.

GD has done acquisition of data, interpretation of data and drafted the manuscript.

EJ carried out the immunoassays and interpretation of data, also performed the statistical analysis.

ShR has made acquisition of data, has been involved in drafting the manuscript.

BD has made acquisition of data, has been involved in drafting the manuscript.

GD have made contributions to conception and design; have been involved in revising it critically for important intellectual content and has given final approval of the version to be published.

All authors read and approved the final manuscript.
